# Aerobic performance in tinamous is limited by their small heart. A novel hypothesis in the evolution of avian flight

**DOI:** 10.1038/s41598-017-16297-2

**Published:** 2017-11-21

**Authors:** Jordi Altimiras, Isa Lindgren, Lina María Giraldo-Deck, Alberto Matthei, Álvaro Garitano-Zavala

**Affiliations:** 1 AVIAN Behavioral Genomics and Physiology, Department of Physics, Chemistry and Biology, Linköping University, Linköping, Sweden; 2 Instituto de Ecología, Universidad Mayor de San Andrés, La Paz, Bolivia; 3 Tinamou Chile S.L, Los Angeles, Chile

## Abstract

Some biomechanical studies from fossil specimens suggest that sustained flapping flight of birds could have appeared in their Mesozoic ancestors. We challenge this idea because a suitable musculoskeletal anatomy is not the only requirement for sustained flapping flight. We propose the “heart to fly” hypothesis that states that sustained flapping flight in modern birds required an enlargement of the heart for the aerobic performance of the flight muscles and test it experimentally by studying tinamous, the living birds with the smallest hearts. The small ventricular size of tinamous reduces cardiac output without limiting perfusion pressures, but when challenged to fly, the heart is unable to support aerobic metabolism (quick exhaustion, larger lactates and post-exercise oxygen consumption and compromised thermoregulation). At the same time, cardiac growth shows a crocodilian-like pattern and is correlated with differential gene expression in MAPK kinases. We integrate this physiological evidence in a new evolutionary scenario in which the ground-up, short and not sustained flapping flight displayed by tinamous represents an intermediate step in the evolution of the aerobic sustained flapping flight of modern birds.

## Introduction

The evolutionary origin of avian flight has been long a matter of scientific debate without unanimous agreement^1,2^. It has been suggested that powered flight appeared as early as in the most ancient true birds such as *Archaeopteryx* ^3^ but this is controversial because early birds experimented with different airborne gliding or flying behaviors^4–6^ and the presence of wings and feathers is not necessarily associated with the origin of flight^7^. In modern birds flight is characterized by wing flapping powered by special antagonistic muscles both housed in a ventrally expanded sternum, but the particular skeletal organization of the pectoral girdle responsible for a true flapping flight evolved only in ornithuromorphs and modern birds^8^. New paleontological and neontological evidence suggest that flapping flight could have evolved in terrestrial birds as a powerful take-off from the ground in a cooperative effort between legs and wings^8–10^. Ultimately, a sustained flapping flight (SFF) became the successful alternative adopted by birds to be subsequently refined by gliding or hovering. SFF is defined here as the ability to maintain flapping flight aerobically without consequences on other physiological functions.

Sustained flapping flight is an energetically costly activity and the cardiovascular system is of crucial importance to support the elevation in metabolism associated with it^11^. Given that heart mass and maximal metabolic rate scale similarly to body mass, it has been suggested that cardiac size limits maximal aerobic scope and, consequently, maximal metabolism during flight^11^. Therefore, our main argument is that evolutionary modifications in the cardiovascular system would have been critical to support the high aerobic demand of sustained flight and that a suitable musculoskeletal arrangement is not enough to prove the sustained flight capabilities of modern birds. Unfortunately soft tissues fossilize poorly and data on cardiovascular variables cannot be directly measured. Thus, the appearance of flapping flight cannot be unequivocally coupled to sustaining aerobic flight unless the cardiovascular system made it possible.

For this purpose we chose several species from the tinamou family because there is some evidence that they have the smallest heart among birds^11^, 0.3% of body mass (average from 9 species), which is several times smaller than in phasianids (0.81% as the average relative heart mass from twenty-two species), which is an ecologically convergent group (summarized graphically in Supplementary Figure 1). Tinamous are the only living flying paleognaths, a clade shared with the flightless Ratites (ostriches, emus, rheas, cassowaries and kiwis). In the evolution of Neornithes or “modern birds”, Palaeognathae split from Neognathae (all the other living birds) in the Cretaceous^12^. Tinamous only perform short and burst flapping flights initiated from the ground^13,14^ and it has been argued that the small heart limits flight^11^.

We devised our study to evaluate the physiological performance of the heart in tinamous; to confirm and unequivocally document the ontogeny of cardiac growth and its adult morphometry; and to provide evidence of putative gene regulatory pathways involved in the architecture of cardiac size. Therefore, we used two tinamou species from the same genus, the Ornate Tinamou (*Nothoprocta ornata*) found in the Andean Highlands of Peru, Bolivia, Argentina and Chile (altitude range 2500–4800 m) and the Chilean Tinamou (*Nothoprocta perdicaria*) found in lowland areas in Chile. These species were compared with Red Junglefowl (*Gallus gallus*) a neognath species with a larger heart mass but with similar short and burst flapping flights initiated from the ground. We also included data from an outgroup species, the American alligator (*Alligator mississippiensis*) to analyze the comparative ontogeny of cardiac growth. Crocodilians are the only extant non-avian archosaurs and have a typical non-avian reptilian small heart^15^.

Our hypothesis was that the relative small mass of the heart in tinamous would restrict their aerobic performance and thermoregulatory capacity, and if the small mass of the heart is phylogenetically related to non-avian archosaurian reptiles, the heart of the Red Junglefowl would be comparatively larger during ontogeny. This multi-level and integrated approach intends to explore proximal and ultimate causes for a morphological trait in living birds that has important implications for the interpretation of the evolution of avian flight.

## Results

**Figure 1:**
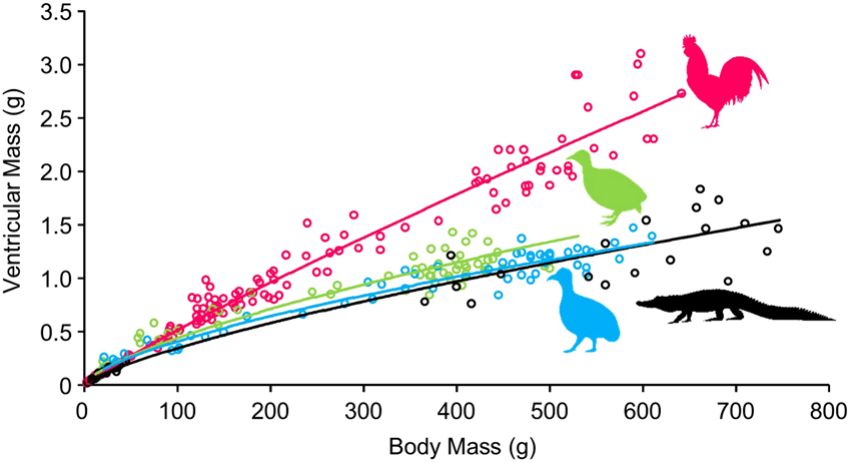
Cardiac growth from embryonic age to juvenile or adult age in Red Junglefowl (*Gallus gallus*) in red, Chilean Tinamou (*Nothoprocta perdicaria*) in green, Ornate Tinamou (*Nothoprocta ornata*) in blue and American alligator (*Alligator mississippiensis*) in black. Power regression lines for each species were obtained after logarithmic transformation and Model II analysis (orthogonal regression) in Minitab 17. The power regression equation are as follows: *Gg*: VM = 0.0085 BM^0.892^; *Np*: VM = 0.0186 BM^0.688^; *No*: VM = 0.0207 BM^0.649^; *Am*: VM = 0.0117 BM^0.737^.

We characterized cardiac growth using power allometric equations (VM = a BM^b^ where VM is ventricular mass and BM is body mass) to the data for each species (Fig. 1). The equations differed significantly in the mass exponent between Red Junglefowl and the tinamous and the alligator. For an individual with a body mass of 700 g, relative ventricular mass would be 0.21% in the alligator and the Ornate Tinamou, 0.24% in the Chilean Tinamou and significantly larger (0.42%) in the Red Junglefowl. The emerging pattern from the data is that cardiac growth in tinamous and alligators follows a similar trajectory while in Red Junglefowl cardiac growth is increased throughout life. The differences are not apparent during early development and the cardiac growth curves start splitting at a body mass approximately above 80 g, which corresponds to an approximate age of 2–4 weeks.

**Figure 2:**
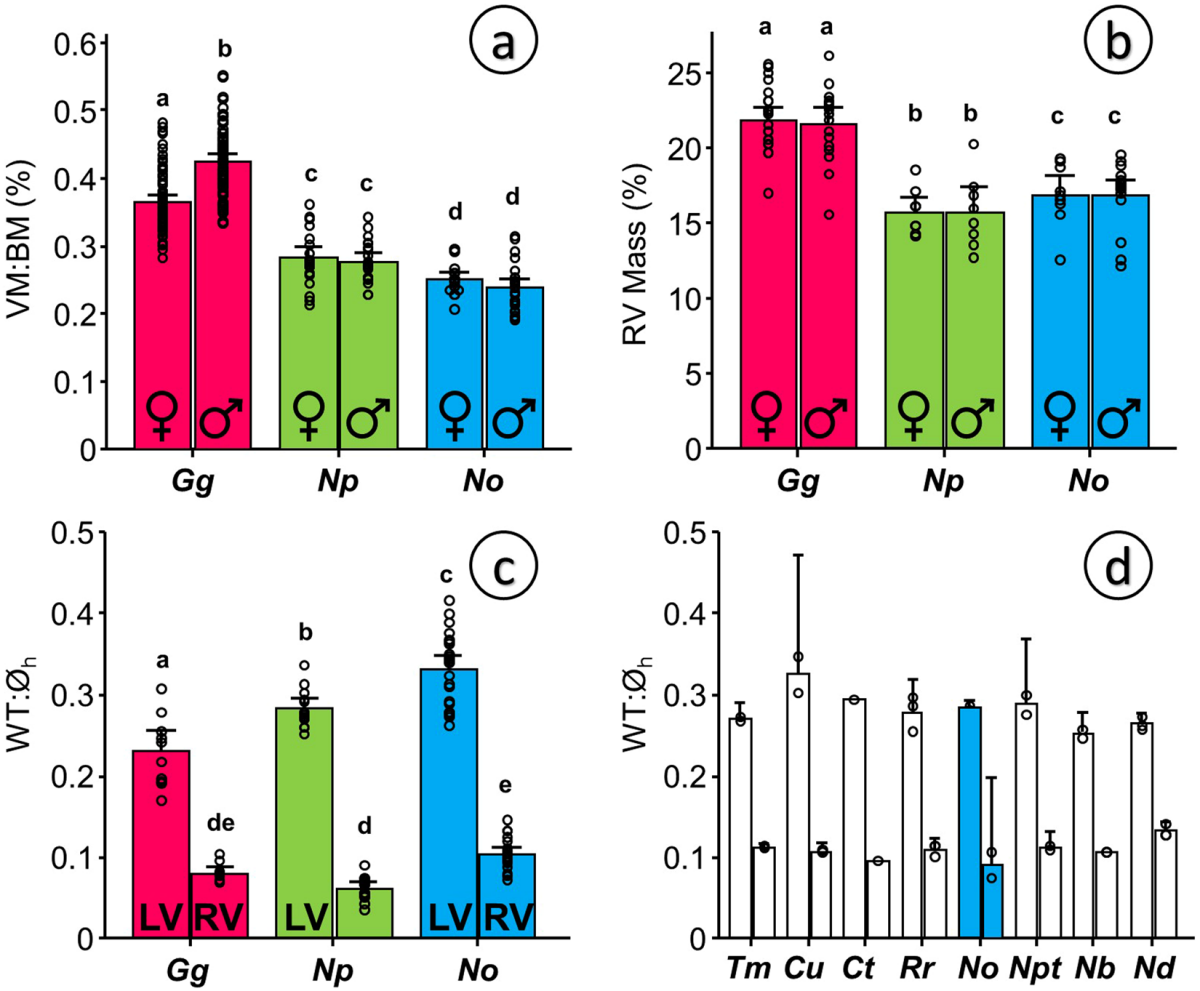
Comparative morphometry of the heart in adult specimens of Red Junglefowl (*Gallus gallus*, *Gg*), Chilean Tinamou (*Nothoprocta perdicaria*, *Np*) and Ornate Tinamou (*Nothoprocta ornata*, *No*). (**a**) Relative ventricular mass in males and females shown as the percentage of ventricular mass to body mass (VM:BM). (**b**) Mass of the right ventricle (RV) in males and females shown as the percentage of RV to VM. (**c**) Left and right ventricular wall thickness normalized to the diameter of the heart (WT:Ø_h_) obtained from the ventricular section showing the attachment of an incipient right atrioventricular valve to the right ventricular free wall (see Material and Methods for details). (**d**) Normalized left and right ventricular wall thickness obtained as in panel C from ethanol preserved specimens of other tinamou species kept at the Colección Boliviana de Fauna at the Universidad Mayor de San Andrés in La Paz, Bolivia. Species nomenclature as in Table 1: *Tm* – *Tinamus major* (N = 2), *Cu* – *Crypturellus undulatus* (N = 2), *Ct* – *Crypturellus tataupa* (N = 1), *Rr* – *Rhynchotus rufescens* (N = 3), *No* – *Nothoprocta ornata* (N = 2), *Npt* – *Nothoprocta pentlandii* (N = 2), *Nb* – *Nothura boraquira* (N = 2), *Nd* – *Nothura darwinii* (N = 3). All data presented as mean and 95% confidence intervals with individual data points shown. N values as follows (in order from left to right in the different panels): a – 68,70,19,21,19,26; b – 21,17,8,8,9,15; c – 10,10,14,14,22,22. For statistical analysis for panels (a), (b) and (c) we used general linear modeling (GLM) considering species and gender or species and ventricle as factors followed by Tukey posthoc test with a customary fiduciary significant level of p < 0.05 (shown as dissimilar letters) in Minitab 17. No statistical analysis was performed for panel d.

Ornate Tinamous had the smallest relative ventricular size of all three species with a ventricular index of 0.24% (SD 0.03, N = 45) followed by Chilean Tinamous (0.28% SD 0.04, N = 40) and Red Junglefowl, with the largest ventricular sizes and a significant difference between males and females (0.42% SD 0.05, N = 70 and 0.36% SD 0.05, N = 68 respectively) as shown in Fig. 2a. The small ventricular mass can be generalized to the entire family Tinamidae regardless of habitat or altitudinal distribution (Table 1). Data on ventricular mass from 13 species from both tinamou subfamilies give a consistently small ventricular mass, ranging between 0.34% in the Small-billed Tinamou *Crypturellus parvirostris* and 0.15% in the Great Tinamou *Tinamus major*. This pattern is clearly evident when accounting for relative heart mass in all the bird species measured to date as compiled by Nespolo and the authors^16^ and graphically shown in Supplementary Figure 1.

**Table 1:** Relative body mass in all tinamou species recorded to date. Altitudinal and latitudinal distribution for each species were obtained from BirdLife International and NatureServe (2014) Bird Species Distribution Maps of the World. The IUCN Red List of Threatened Species. Version 2016-2. (http://maps.iucnredlist.org/index.html).

Species	Common name	Body mass (g)	%VM:BM	Altitudinal distribution	Latitudinal distribution
**Subfamily Tinaminae (forest dwelling tinamous)**					
*Nothocercus bonapartei*	Highland Tinamou	885.2	0.194^1^	500–2500 m	11°02’N – 05°21’S
*Tinamus major*	Great Tinamou	1169.4	0.147^1^	0–1500 m	18°55’N – 18°04’S
		1140.0	0.172^5^		
*Crypturellus soui*	Little Tinamou	233.0	0.194^1^	0–2000 m	19°07’N – 22°30’S
*Crypturellus undulatus*	Undulated Tinamou	537.5	0.187^5^	0–900 m	08°24’N – 27°47’S
*Crypturellus parvirostris*	Small-billed Tinamou	136.8	0.341^4^	0–1200 m	00°23’S – 28°36’S
*Crypturellus tataupa*	Tataupa Tinamou	275.0	0.192^5^	0–1400 m	02°20’S – 31°35’S
**Subfamily Rhynchotinae (open fields dwelling tinamous)**					
*Rhynchotus rufescens*	Red-winged Tinamou	821.7	0.198^3^	0–2500 m	03°26’S – 41°10’S
		796.6	0.190^5^		
*Nothoprocta ornata*	Ornate Tinamou	505.0	0.235^4^	2500–4800 m	07°40’S – 30°00’S
		500.0	0.224^5^		
*Nothoprocta perdicaria*	Chilean Tinamou	395.5	0.284^4^	400–2000 m	28°24’S – 41°46’S
*Nothoprocta pentlandii*	Andean Tinamou	258.0	0.298^5^	1500–4000 m	02°50’S – 36°54’S
*Nothura boraquira*	White-bellied Nothura	310.0	0.178^5^	0–500 m	03°12’S – 22°43’S
*Nothura darwinii*	Darwin’s Nothura	251.4	0.280^4^	1000–4300 m	09°24’S – 44°29’S
		197.0	0.321^5^		
*Nothura maculosa*	Spotted Nothura	275.0	0.265^2^	0–2300 m	05°06’S – 44°18’S

^1^From Hartman, F.A., 1961. Locomotor mechanisms of birds. Smithsonian Miscellaneous Collections 143, 1–91.

^2^From Dorst, J., 1972. Poids relatif du coeur chez quelques oiseaux des hautes Andes du Perou. L’oiseau et la revue française d’ornithologie 42, 66–73.

^3^From De La Riboisiere, J., 1910. Recherches organométriques en fonction du régime alimentaire sur les oiseaux, Faculté des Sciences. Université de Paris, Paris.

^4^Own data from fresh specimens.

^5^Own data from specimens preserved in ethanol.

Relative right ventricular mass was also highest in Red Junglefowl (21.8% SD 2.3, N = 38) than in both tinamous (16.9% in Ornate Tinamou and 15.7% in Chilean Tinamou) as shown in Fig. 2b. Despite having smaller hearts, the left ventricular wall was thicker in tinamous than in Red Junglefowl (Fig. 2c). In Red Junglefowl, the left ventricular wall accounted for 23% (SD 4, N = 10) of the diameter of the heart and this was larger in the Chilean Tinamou (25%, SD 2, N = 14) and even larger in the Ornate Tinamou (33%, SD 5, N = 22). These values are comparable to those obtained in ethanol preserved hearts from other tinamou species, which ranged from 25% in the White-bellied Nothura *Nothura boraquira* to 32% in the Small-billed Tinamou *Crypturellus parvirostris* (Fig. 2d).

**Figure 3:**
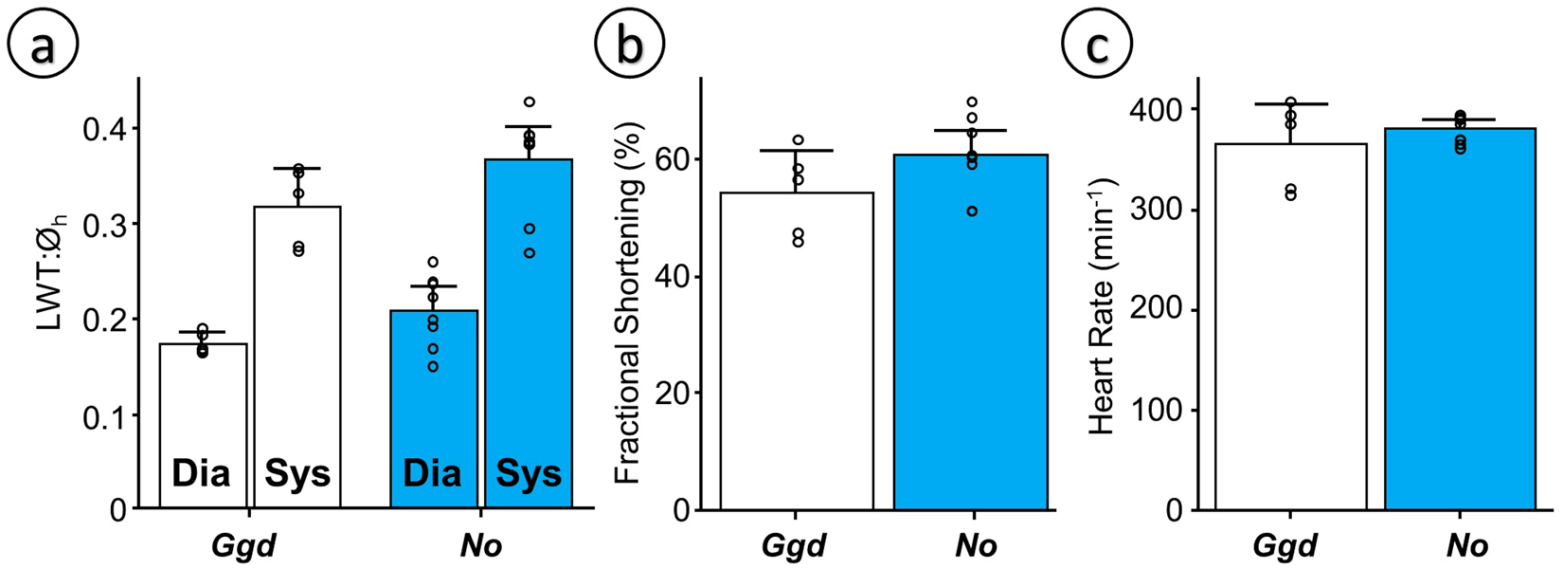
Functional echocardiographic measurements of the heart in conscious birds placed in supine position under tonic immobility. Bantam chickens (*Gallus gallus domesticus*, *Ggd*) were used for comparison with the Ornate Tinamou (*Nothoprocta ornata*, *No*). (**a**) Left ventricular wall thickness normalized to the diameter of the heart (LWT:Ø_h_) from a parasternal echocardiographic plane in which the right ventricular free wall is incipient but without a visible right ventricular chamber (see Material and Methods for details). (**b**) Fractional Shortening (%) of the cardiac muscle at the same plane. (**c**) Heart rate estimated from the time between subsequent peak systolic events in M-mode echo. All data presented as mean and 95% confidence intervals with individual data points shown (N = 5 for *Ggd* and N = 8 for *No*). Due to small sample size and an assumed lack of normality and homocedasticity of the data, permutation tests were used to test for differences between species using StatBoss (see Material and Methods for further details). No significant differences were found.

**Figure 4:**
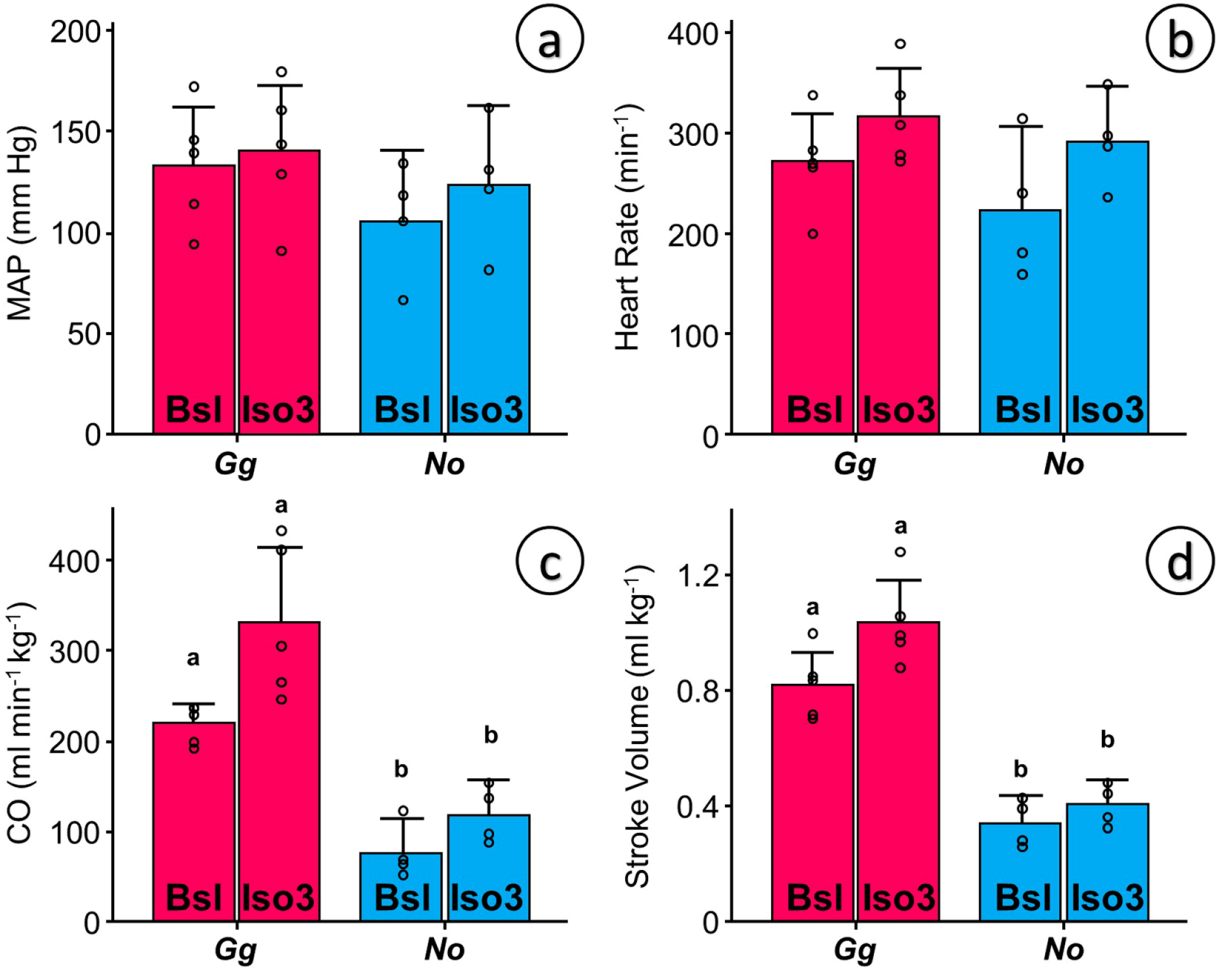
Functional measurements of cardiovascular variables in ketamine-xylazine anesthetized Red Junglefowl (*Gallus gallus*, *Gg*) and Ornate Tinamou (*Nothoprocta ornata, No*) before (Bsl – baseline) and after the administration of 3 ug kg^−1^ of isoproterenol (Iso3). (**a**) Mean Arterial Pressure (MAP, mm Hg) measured from an intravascular catheter in the brachial artery. (**b**) Heart Rate calculated from the instantaneous pressure trace. (**c**) Mass specific total Cardiac Output (CO) estimated from a transit flow probe placed in the aortic arch after the splitting of the brachiocephalic arteries (see Material and Methods and Suppl. Figure 5 for details) and (**d**) Stroke Volume calculated from the quotient between CO and heart rate. All data presented as mean and 95% confidence intervals with individual data points shown (N = 5 for *Gg* and N = 4 for *No*). Due to small sample size and an assumed lack of normality and homocedasticity of the data, paired permutation tests were used to test for differences between species and for the effect of isoproterenol treatment using StatBoss (see Material and Methods for further details). A customary fiduciary significant level of p < 0.05 was used after compensation for multiple comparisons. Statistical differences between species but not due to treatment were seen only in panels (c) and (d) and are shown by dissimilar letters.

Measurements of wall thickness using echocardiography in conscious tinamous are comparable to the morphometric measurements. They are not equivalent because they were not performed at the same anatomical landmark. Left ventricular wall thickness was not significantly different between Ornate Tinamous and bantam chickens (21% in the tinamou vs. 17% in the bantam chicken (p = 0.08) in diastole; 37% vs. 32% respectively (p = 0.12) in systole, Fig. 3a). Fractional shortening in conscious animals (Fig. 3b) and heart rate in conscious (Fig. 3c) or anesthetized animals (Fig. 4b) did not differ either and mean arterial pressure was comparable between Red Junglefowl and Ornate Tinamou (Fig. 4a). Mean arterial pressure was 115 mmHg (SD 30) in Ornate Tinamou and 136 mmHg (SD 30) in Red Junglefowl. Heart rate was 259 beats per minute (SD 65) in Ornate Tinamou and 295 beats per minute (SD 52) in Red Junglefowl. Isoprenaline, a beta-adrenergic receptor agonist, at a dose of 3 ug kg^−1^ did not significantly stimulate cardiac function but a highly significant difference in cardiac output and stroke volume was observed (Fig. 4c,d). Cardiac Output was only 98 ml min^−1^ kg^−1^ (SD 37) in Ornate Tinamou. It was 2.8 fold larger in Red Junglefowl (276 ml min^−1^ kg^−1^, SD 84). Stroke volume was 0.37 ml kg^−1^ (SD 0.08) in Ornate Tinamou and 2.5 fold larger in Red Junglefowl (0.93 ml kg^−1^, SD 0.17). Altogether, the results point out that the small size of the tinamou heart limits cardiac output but not the capability of the heart to generate pressure. Hematology values are summarized in Supplementary Table 1. A clear difference between males and females was observed in the Red Junglefowl but not in the tinamou species.

**Figure 5:**
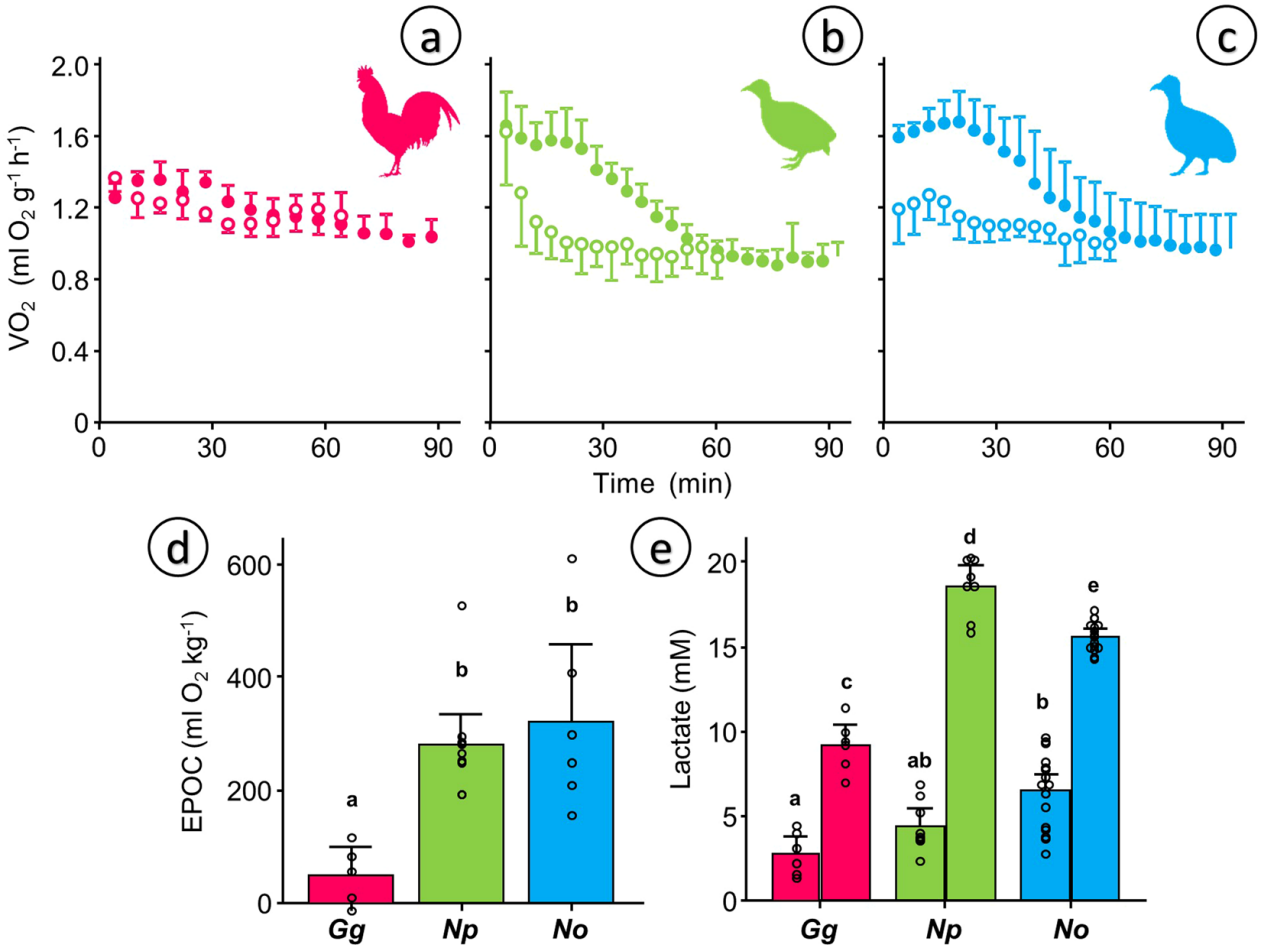
Metabolic measurements before and after a 3 min chase-and-exhaust protocol. ABC) Mass-specific oxygen consumption (VO_2_) in adult individuals of (**a**) Red Junglefowl (*Gallus gallus*, *Gg*, N = 5), (**b**) Chilean Tinamou (*Nothoprocta perdicaria*, *Np*, N = 10) and (**c**) Ornate Tinamou (*Nothoprocta ornata*, *No*, N = 6). Open symbols show the data for the 60 min baseline measurements and closed symbols show the data for the 90 min following the chase protocol. (**d**) Excess post-exercise oxygen consumption (EPOC) obtained by integrating the pre- and post-curves shown as ABC. (**e**) Plasma lactate levels obtained using the same protocol in a separate group of individuals (*Gg* N = 6, *Np* N = 8, *No* N = 16). Data in A–C presented as mean and standard deviations. Data in D–E presented as mean and 95% confidence intervals with individual data points shown. For statistical analysis we used general linear modeling (GLM) considering species (D) and species/treatment (E) as factors followed by Tukey posthoc test with a customary fiduciary significant level of p < 0.05 (shown as dissimilar letters) in Minitab 17. No statistical analysis was performed for panels ABC because the integrated response is considered in panel (d).

Resting metabolic rate 70–90 min after an aerobic challenge did not differ between species: 1.05 mlO_2_ g^−1^ h^−1^ (SD 0.07 N = 5) in Red Junglefowl (Fig. 5a) vs. 0.91 mlO_2_ g^−1^ h^−1^ (SD 0.07 N = 10) in the Chilean Tinamou (Fig. 5b) and 0.98 mlO_2_ g^−1^ h^−1^ (SD 0.18 N = 6) in the Ornate Tinamou (Fig. 5c) but excess post-exercise oxygen consumption (EPOC) was almost 6-fold larger in both tinamou species. EPOC averaged 297 mlO_2_ kg^−1^ in tinamous and was only 50 mlO_2_ kg^−1^ in Red Junglefowl (Fig. 5d). This is also reflected in the lactate values reached after the aerobic challenge (Fig. 5e), which were significantly higher in tinamous (18.7 mM SD 1.7, N = 8 in Chilean Tinamou and 15.7 mM SD 0.9, N = 16 in Ornate Tinamou) than in Red Junglefowl (9.2 mM SD 1.5, N = 6).

**Figure 6:**
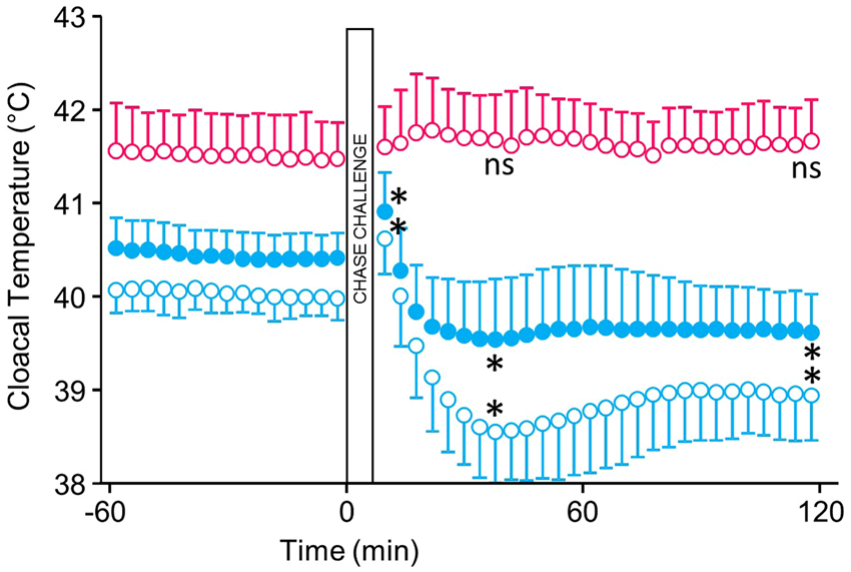
Cloacal temperature before and after a 3 min chase-and-exhaust protocol in Ornate Tinamou kept at an ambient temperature of 4 °C (open blue symbols) and 25 °C (closed blue symbols). The chase-and-exhaust protocol was carried out after a baseline measurement lasting 1 h. For comparison, data on cloacal temperature in bantam chickens kept at 4 °C (open red symbols) that underwent the same protocol are shown. Data from chickens at 25 °C did not differ substantially and is shown in Suppl. Figure 2. All data presented as mean and standard deviations (N = 6 for both species). Paired permutation tests were used to compare baseline temperatures preceding the chase-and-exhaust protocols (120 min) with the temperatures 30 min after the test. Significant differences were observed only for the Ornate Tinamou (p = 0.03 in both cases) and are shown by “*” in the graph. “ns” indicate no significant difference.

Cloacal temperatures were more labile in the Ornate Tinamou than in the Red Junglefowl and increased significantly after the chase-challenge (Fig. 6). In the colder environment (5 °C) cloacal temperature was lower than in the warmer thermoneutral environment (25 °C) only in the tinamou, 39.9 °C (SD 0.2 N = 6) vs. 40.4 °C (SD 0.3 N = 6). Forty minutes after the chase-challenge cloacal temperature had dropped significantly by 1 °C at both thermal environments, to 38.5 °C (SD 0.5 N = 6) at 5 °C and to 39.5 °C (SD 0.6 N = 6) at 25 °C, and body temperature remained low even 2 h after the challenge. The lability in body temperature was not seen in Red Junglefowl (Fig. 6 and Suppl. Figure 2).

**Figure 7:**
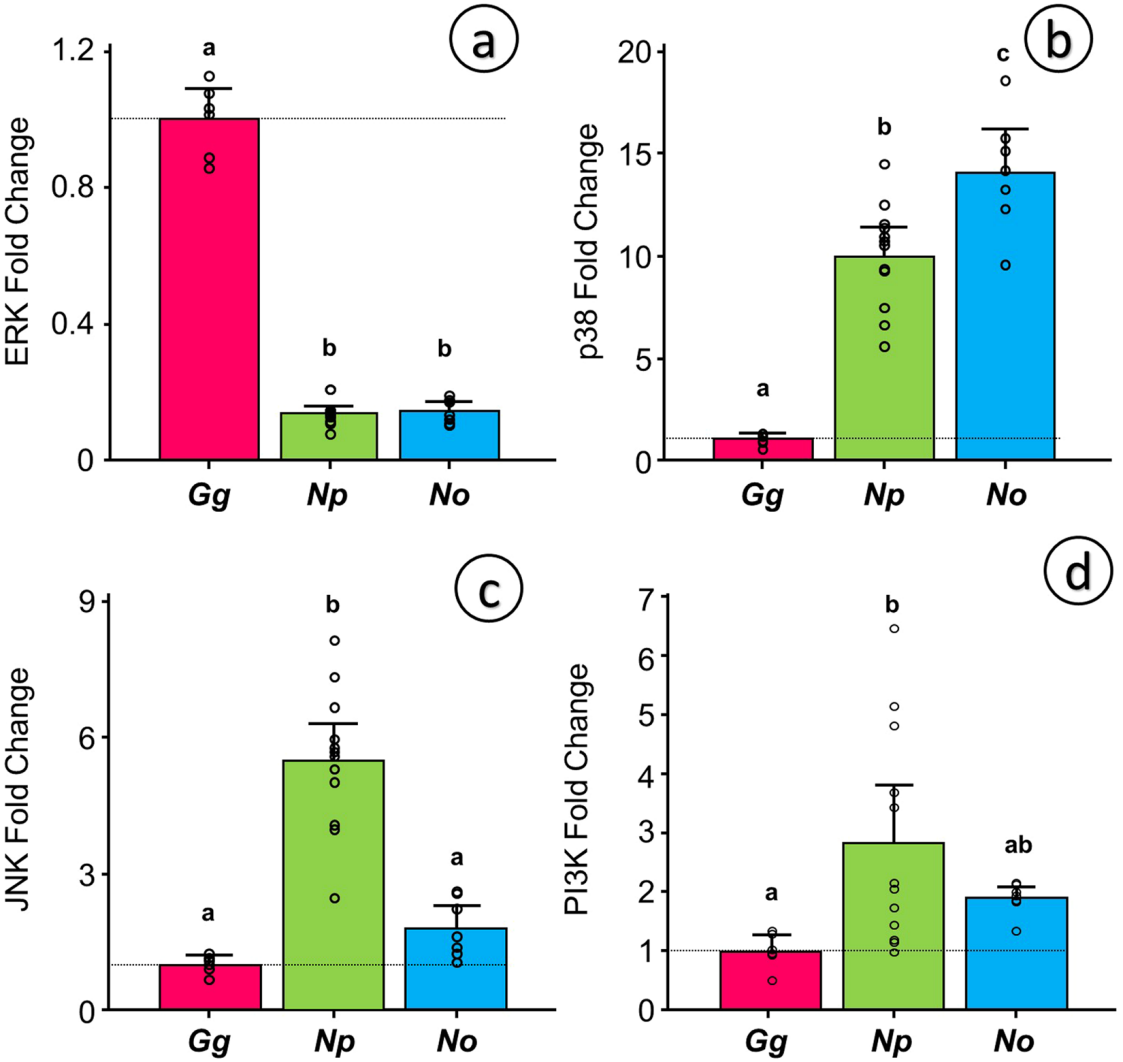
Relative expression of the three main MAP kinase genes: ERK (**a**), p38 (**b**) and Jnk (**c**) and PI3K (**d**) in Red Junglefowl (*Gallus gallus*, *Gg*, N = 6), Chilean Tinamou (*Nothoprocta perdicaria*, *Np*, N = 12) and Ornate Tinamou (*Nothoprocta ornata*, *No*, N = 7). All values calculated in relation to the expression in Red Junglefowl after normalization against three housekeeping genes: GAPDH, β-actin and TBP. Dotted line indicates the reference expression level for Red Junglefowl. All data presented as mean and 95% confidence intervals with individual data points shown. For statistical analysis we used general linear modeling (GLM) considering species as a factor followed by Tukey posthoc test with a customary fiduciary significant level of p < 0.05 (shown as dissimilar letters) in Minitab 17.

To further explore the ventricular mass differences we focused on the gene expression of four kinases in the PI3K/Akt and MAPK signaling pathways: ERK (gene *ERK2*), JNK (gene *JNK1*), p38 (gene *p38*) and PI3K (gene *PIK3CA*). We found a consistent downregulation in *ERK2* (Fig. 7a) and an upregulation in *p38* (Fig. 7b) in both tinamou species in relation to the Red Junglefowl. *ERK2* was downregulated down to 14% of the Red Junglefowl values in both species and *p38* was upregulated 10-fold and 14-fold in the Chilean Tinamou and the Ornate Tinamou respectively. *JNK1*, on the other hand, was significantly 5.5-fold upregulated in the Chilean Tinamou, but only 1.8-fold upregulated in the Ornate Tinamou not reaching statistical significance (Fig. 7c). No significant differences were found for *PIK3CA* (Fig. 7d).

## Discussion

### The tinamou heart can generate pressure but not flow

Our results confirm that the heart of tinamous is the smallest among all extant bird species^11,16^. This is true for all thirteen tinamou species studied to date (by us or by others) and the inter-specific variation is not related to altitudinal or latitudinal geographic distribution, habitat or their belonging to the subfamily Tinaminae or Rhynchotinae (Table 1). The small ventricular mass inevitably limits its functional output and mass specific cardiac output and stroke volume in the Ornate Tinamou to less than half the chicken values (Fig. 4c,d), the values obtained in neognathous species^17^, and even in other palaeognathous birds such as the emu^18^. Such low stroke volumes (0.34–0.4 ml min^−1^ kg^−1^) are comparable to those in alligators^19^ but the faster heart rates in tinamous account for five-fold larger cardiac outputs.

Application of Fick’s principle to the cardiac output and hematology data allows for an estimation of maximum oxygen consumption of 1.32 ml O_2_ g^−1^ h^−1^ for the Ornate Tinamou and 3.61 ml O_2_ g^−1^ h^−1^ to the Red Junglefowl. The estimate for the Ornate Tinamou is below our *in vivo* measurements of 1.62 ml O_2_ g^−1^ h^−1^. Even the estimate for the Red Junglefowl is lower than the maximal metabolic rates measured in the species (6.4 ml O_2_ g^−1^ h^−1^ and 4.1 ml O_2_ g^−1^ h^−1^ in males and females respectively^20^). The discrepancy is most likely due to the cardiodepressive effects of anesthetics already documented in birds^21,22^. This means that the cardiac outputs reported in the study, albeit comparable between species, are more than 20% lower than the expected *in vivo* values.

Cardiac output is limited but cardiac contractility is not compromised. Fractional shortening is high and comparable to equivalent measurements in bantam chickens (Fig. 3b) and White Leghorn chickens^23^. The left ventricle of the Ornate Tinamou is competent to generate bird-like mean arterial pressures (above 120 mmHg, Fig. 3a) while maintaining bird-like heart rates (above 290 min^−1^ during anesthesia and above 380 min^−1^ in conscious animals, Figs 4b and 3c respectively).

The novel observation of crocodilian-like stroke volumes and reduced cardiac outputs combined with normal arterial pressures is a key finding for a bird species and provides the first clue of the ancestrality of tinamous. We sustain that the tinamous achieved a high systemic pressure taking advantage of the thickening of the left ventricle (Fig. 2c) without an enlargement of the cardiac chambers. Because of the small chamber size, a modest thickening of the left ventricular wall would facilitate the development of higher pressures without incurring in excessive wall tensions as predicted by the principle of Laplace^24^.

### The cardiac morphology and physiology of the tinamous limits aerobic performance

Ventricular size and its flow-limited capability have no effect on resting metabolism in thermoneutral conditions. Oxygen consumption of the Ornate Tinamou and the Chilean Tinamou is not different from the measures in Red Junglefowl under similar experimental conditions (close to 1 ml O_2_ g^−1^ h^−1^). Our values for Chilean Tinamous are 32% higher than those obtained previously^25^, but they are more robust considering sample sizes and the fact that there was an agreement between the two tinamou species. The values contrast with the lower resting metabolism of Ratites, which is likely due to the reduction in pectoral muscle and the loss of flight instead of phylogeny, as pointed out in large interspecific studies of metabolism^26^. Our metabolic measurements are consistent with the fact that tinamous have well developed pectoral muscles^27^, with percentage values of pectoral mass ranging between those in many other bird families as shown in Supplementary Figure 3.

Albeit not in resting conditions, the small heart in tinamous is a relevant hindrance when demand for oxygen supply increases. When both tinamou species are challenged to fly and perform above resting levels, they reach exhaustion quickly while significantly accumulating lactate because during this period the heart is unable to supply enough oxygen to the body. Large increases in lactate are also observed in restrained estuarine crocodiles with slow recoveries^28^. Aerobic metabolism after the exhaustion challenge, the so called post-exercise oxygen consumption (EPOC)^29^ was also remarkably long-lasted (60 min) for tinamous but almost absent for Red Junglefowl, despite the fact that lactate also increased in the latter. Lactate oxidation is not the main metabolic burden post-exercise^30^ so the main conclusion from our results is that the larger lactate release and the significant EPOC response reflect that the exhaustion challenge requires an anaerobic contribution that is also considerably longer than what is seen in mammals (17 min in sprint wheel running in mice for example^31^). From a comparative point of view, the tinamou EPOC could be considered similar to the response in lizards^32^ and we infer that the cardiac performance of tinamous closely approximates, in some regards, that of non-avian archosaurs (namely crocodilians) and it may be that this cardiac performance is the ancestral condition for birds.

The aerobic challenge also imposed limitations to the ability to regulate body temperature. Basal cloacal temperatures in the Ornate Tinamou are clearly avian-like albeit on the lower part of the range^33^. Body temperature drops below resting values for a period longer than 2 h and the effect is enhanced at lower ambient temperatures. The striking heterothermic swing (rise in body temperature during exhaustion and drop thereafter) is likely magnified by the experimental conditions where the animals could not use behavioral thermoregulation but it was not seen in bantam chickens, which attests to the physiological difference between species. To distinguish if this observation is an adaptive energy-sparing mechanism or a maladaptive consequence of hyperventilation-driven body cooling further studies are needed, but the body temperature of the Ornate Tinamou clearly shows greater variations than chickens and also a larger dependence on ambient temperatures.

It is important to emphasize that the physiological limitations ascribed to a smaller heart mainly apply to challenging scenarios such as the exhaustive bouts of exercise imposed experimentally or the explosive burst of flight experienced in the wild when escaping from potential predators, which could be the most significant aerobic challenges a tinamou is exposed to attending to the EPOC measured^32^. In resting conditions tinamous are not metabolically challenged and remain thermally stable. The flight performance in tinamous is highly conditioned by their small heart and this could explain why tinamous avoid flying as much as possible. Tinamous prefer to escape from predators walking and running and use their cryptic plumage and secretive habits as the main antipredatory mechanism. When they do not have any other option but flying, they do it in a burst-like manner with a powerful jump. Such flapping flight, normally accompanied with a strident vocalization, can be performed two or three consecutive times before the bird is unable to fly again and it is possible to hand-catch them^13,34^. This is what we define as non-sustained flapping flight (NSFF) in the study and differs from the burst flight of phasianids.

### The ontogenic development of the tinamous’ heart show a clear signal of ancestrality

The ontogenic development of the heart of both tinamou species differs widely from that of the Red Junglefowl but otherwise is remarkably alike that of the alligator. Estimated relative ventricular mass at adulthood is 0.21–0.24%, similar to alligator published values^15,35^. Thus, we speculate that the heart in neognathous birds display a larger proliferative activity that would yield a bigger heart. The similarity between cardiac growth between tinamous and alligators but not Red Junglefowl is hard to ascribe to a casual or convergent process, and the most parsimonious interpretation is that the neognathous larger heart is an evolutionary novelty in comparison to the tinamou heart that is more similar to non-avian reptilian species and could be assumed as ancestral. In fact, from a compiled list of nineteen species (3 turtles, 7 snakes, 7 lizards and 2 crocodiles), all but two snake species have a relative heart mass below 0.35%^36^.

The genetic mechanisms behind the differential cardiac growth in bird species are mostly unknown so we singled out PI3K/Akt and MAPK pathways as a first approach because of their prominent role in cardiac development and plasticity in mammals^37,38^. MAPK activation, mainly ERK, also regulates cardiogenesis and differential cell lineage growth in the embryonic chicken heart^39^. The expression pattern that we report for the three terminal MAPKs and for PI3K is consistent with the knowledge in mammalian myocardial tissue^37^. We speculate that the observed ERK upregulation in Red Junglefowl promotes cardiac growth resulting from growth-factor mediated physiological hypertrophy^37^. On the other hand, the stress-activated kinases, p38 and JNK, are either upregulated or do not change in tinamous, which is congruent with the antagonistic effects reported for both JNK^40^ and p38^41^ on mammalian cardiac growth. Altogether, a reduced ERK expression and an enlarged JNK/p38 expression of tinamous may be a signature of a poor cardiac proliferative capability in primitive birds, speculations that require further studies.

### The “heart to fly” hypothesis on the acquisition of sustained flapping flight in Neornithes: “sustained flapping flight requires a large heart for the aerobic performance of the flight muscles”

Based on all our evidence, we propose that the small heart mass in tinamous is a plesiomorphic trait for all Neornithes, a trait which was likely shared with archosaurian ancestors. The lack of fossil evidence for dinosaurian or ancestral avian hearts prevents direct inferences as to their size but the evidence that several avian characteristics were acquired progressively along avian evolution, for instance a unidirectional air ventilation and high metabolic rates^42^, supports our interpretation that novel metabolic demands imposed a strong adaptive pressure on the basal archosaurian heart to increase blood systemic pressure and metabolic rates and to perform some kind of flapping flight, more like a NSFF as the first step.

Support for the plesiomorphy of the small heart comes from the fact that among neognathous birds, no reduction of the flight capacity has been associated with a reduction in heart mass^16^ despite the fact that pectoral muscle mass is actually reduced, as consistently shown in island bird lineages undergoing transitions to secondary flightlessness^43^.

Nevertheless, we cannot discard the alternative evolutionary scenario in which the small tinamou heart derived from a larger heart in ancestral species with good flight capabilities. For example, some authors consider that an enlarged heart mass could have appeared with the onset of endothermy which presumably preceded the onset of flight^44,45^.

But if a small heart and NSFF are derived characters instead of ancestral, what selection pressures would have been in operation? Note that tinamous need to constantly use cryptic behaviors that restrict their foraging and reproduction niches, they are unable to use flight for migration or dispersion, and they are highly susceptible to predation when exhausted. None of these seems to favor a positive selection pressure for a secondary acquisition of a small heart. Not even the selection pressure of decreased oxygen gradients at altitude triggered cardiac enlargement in high altitude tinamous such as the Ornate Tinamou.

Along the phylogenetic history of birds, the acquisition of a well-developed pectoral musculature (inferred from osteological characters, *i.e*. the progressive increase of the carina in the sternum) has been directly coupled to the capacity for powered flapping and sustained flight, and some authors have even ventured that birds without carina and pectoral musculature such as *Archaeopteryx* could have displayed powered flapping flight^3,46^. Our physiological data does not support the claim that avian species preceding Neornithes would have been capable of sustained flapping flight (SFF). Ruben actually proposed that an anaerobic reptile-like mode of muscular performance would be enough to let *Archaeopteryx* to take flight from the ground and to have a powered flapping flight^46^. We provide evidence that a high aerobic capacity is required for SFF based on the fact that tinamous are metabolically limited and cannot perform SFF.

Tinamous are ground-dwelling birds that are able to take off from the ground and display NSFF^13,34^. All the osteological and muscular machinery of tinamous belong to a Neornithine flying bird, but our results show that even in the presence of a big carinated sternum that supports enough pectoral musculature (Supplementary Figure 3), a small heart limits aerobic scope and is not compatible with SFF.

The evolutionary way in which the “modern bird flight” appeared in the avian lineage from archosaurian ancestors has been heavily debated for decades^1,2^. The trees-down flight hypothesis considers that the powered flight capacity was acquired from arboreal gliding animals. In accordance with this point of view flapping flight would have preceded the ability to initiate flight from the ground. An alternative hypothesis proposes that powered flight first required the capacity to take up the flight from the ground. Our hypothesis fits better with the second point of view and is not in conflict with the fact that several fossil non-Neornithes avian lineages would have been arboreal or gliders because animals without an adequate aerobic physiological capacity could have taken the advantage of climbing trees or reaching other elevated points in order to glide but not to perform a SFF. In this situation the control of gliding and landing could explain the early evolution of complex wings and feathers, including the presence of the alula^47^.

With the evidence that the cardiac morphology and physiology of tinamous is not suited for sustained flight we propose a new hypothesis on the acquisition of flight in Neornithes. The hypothesis, which we call the “heart to fly” hypothesis states that sustained flapping flight requires a large heart for the aerobic performance of the to the flight muscles. In consequence, the NSFF of tinamous is ancestral and represents the intermediate flight strategy between the non-Neornithes avian fossils and the modern neognathes. In this scenario, the typical jumping take-off from the ground followed by the NSFF that tinamous perform today is a reflex of the putative first type of flight performed by Neornithes, point of view that is in agreement with the neontological-based proposals of the evolution of the avian flight^6,10,48^. Notice that although phasianids display a similar flight mode this is not reflected in an exhaustion of aerobic reserves and, from our definition, it is classified as SFF and not as NSFF.

Based on the new hypothesis we envision the evolutionary history of modern birds as two different paths from an ancestor with a small heart and NSFF, much like the extant tinamous. The first path is the one exemplified by Ratites, in which the loss of the flight capacity (including the loss of all the pectoral musculature and their skeletal support) was aimed at optimizing cursorial abilities for better foraging and predator evasion. The second path is the one followed by Neognaths in which SFF was possible because of the larger heart and the suitable musculoskeletal organization were already in place. Only at this point, the adequate increase in stroke volume and cardiac output allowed for the real conquest of the air for modern Neognaths, and from here the evolution of other types of flight as gliding, soaring, hovering and even returning to short flapping flights and flightlessness.

Two potential conflicts with the “heart to fly” hypothesis require further discussion, namely the larger heart mass of Ratites and the assumed flight capabilities of Lithornitids, an extinct paleognath clade.

First, the heart in the few Ratites species studied to date, i.e. Ostrich, Emu and Greater Rhea is not small and fall in the range of most Neognath families (Suppl. Figure 1). Because Ratites do not fly our “heart to fly” hypothesis implicitly requires that cardiac enlargement in birds occurred independently more than once. For ratites the selective pressure could have been the need for running endurance. Ostriches, for example, are acknowledged as the fastest bipeds with the largest capacity for long-endurance running^49^. Emus, on the other hand, have aerobic scopes in the range of 11–36 times basal metabolic rates while running^18,50^, values that are higher than the aerobic scopes measured in flying birds^50,51^. Based on their nocturnal lifestyle and sedentarity, it is tempting to speculate that kiwis may have retained an ancestral small heart but data is missing.

Although all modern phylogenies support the early divergence of paleognaths in the evolution of Neornithes, there are differences in the proposed phylogenetic relationships between ratites and tinamous. Morphological phylogenies support an early divergence of tinamous followed by ratites as a derivate monophyletic group^52,53^. This scenario is in line with our hypothesis of the plesiomorphy of tinamou traits and implies the secondary loss of flight in ratites. Molecular phylogenies, however, nest tinamous inside ratites^54–58^. At first sight, this could imply that tinamous acquired flight secondarily from flightless ancestors but we find it highly unlikely. Our suggestion, which is compatible with molecular phylogenies, is that the ancestral paleognath was capable of flight (a NSFF type) and lost it multiple times in different Ratite lineages^59^. Living tinamous, descendants of some line of flying paleognaths, conserved NSFF and their ancestral cardiovascular traits.

The second potential conflict relates to the flight capabilities of extinct paleognaths, which is inferred from the dispersed geographical location of fossil findings. The more recent molecular calibrations date the origin of Neornithes and the splice of Palaeognathae and Neognathae in the Late Cretaceous^58^, estimations congruent with the scarce fossil record of this period^12^. Although neognath fossils are best preserved^60^, fragmentary postcranial material of *Iaceornis* ^61^ could represent the earliest palaeognath present in the Late Cretaceous^12^, but no solid fossil evidence of a Cretacic palaeognath with NSFF exists. The first paleognath fossils are from the Paleocene (60–66 million years ago) and correspond to flightless ratites from Europe and South America^62,63^. Tinamous appear in the fossil record much later, in the early Miocene, 16.5 million years ago^64^. The best preserved non-Ratite palaeognathae fossils are the medium-sized volant Lithornithiforms known from the Paleocene and Eocene layers of North America and Europe^65,66^. They are taxonomically rooted with all modern birds, either as a sister taxa of all the Neornithes^52,66^, as sister taxa of all other paleognaths^59^ or as a sister taxa of tinamous^53,64,67^. Based on morphological traits from fossil findings, Lithornithiformes were described as capable of sustained flight^65^, and this suggestion was later used to justify the outcomes of recent molecular paleognath phylogenies that place for example New Zealand kiwis as the closest relatives of Madagascar elephant birds, or South American tinamous clustering with New Zealand moas^56,57,59^. Counter to this argument, the distribution of several tetrapod fossils is congruent with the presence of ephemeral land bridges in the Late Cretaceous between continental land masses^68^ and geologic evidence show that it was possible^69^. In consequence, it is possible that ancient palaeognath birds with tinamou-like cardiovascular physiology and NSFF dispersed widely in Late Cretaceous and Early Cenozoic by walking^70^. This could explain the known distribution of fossil and extant palaeognath species in agreement with our hypothesis.

Our results highlight the crucial importance of physiology, specifically cardiac physiology to understand more completely the evolution of the avian flight. The mechanical and aerodynamic interpretations inferred from the fossils need to consider that this machinery needs a power source, the heart, and that final flight performance depends on it. Although our “heart to fly” hypothesis cannot be unequivocally proven we propose that the physiological characteristics of tinamous are better explained as plesiomorphic traits rather than being secondarily derived. Distinguishing between the two evolutionary scenarios will require combined efforts from paleontologists, biomechanicists and comparative physiologists.

## Methods

### Animals

Adult Ornate Tinamous *Nothoprocta ornata* for the physiological experiments were born in captivity from a founding group of captured wild birds or artificially incubated wild eggs obtained in the surroundings of the town of Qurpa, Bolivia (3800 meters above sea level). Animals were hold at the animal facilities in the Cota-Cota campus (3420 m, Universidad Mayor de San Andrés, UMSA, La Paz, Bolivia) in 8 m^2^ pens holding up to 5 animals per pen and exposed to natural conditions. Animals were fed *ad libitum*. Adult and juvenile Ornate Tinamous for the anatomical studies were hunted with shotgun mainly during the dry season (May to July) in several localities in the Bolivian high plateau between 3800 and 4300 m. Hunting and animal maintenance were carried under the permission for scientific studies from the Bolivian General Direction of Biodiversity and Protected Areas (DGBAP). Chilean Tinamous *Nothoprocta perdicaria* were obtained from Tinamou Chile SL, a farm located in the city of Los Angeles (140 m, VIII Región del Bío-Bío, Chile). Adult individuals at the farm were kept in mixed reproductive groups in 12 m^2^ pens. Young individuals were kept in reduced groups (up to 10 individuals) in 0.5 m^2^ square holding boxes under tungsten filament bulbs used for heating. Adult Red Junglefowl *Gallus gallus* were kept at the research chicken house of the University of Linköping (Ljungsbro, Sweden, 71 m) in 22 m^2^ indoor pens at 19 °C and under 12:12 h light:dark cycle. Food was provided *ad libitum*. The population has been kept under non-selected captive conditions since 1993. Young individuals were kept at the hatchery on the university campus in individual pens at 28 °C and under 12:12 light:dark cycle with food provided *ad libitum*.

Adult domesticated chickens of a bantam breed were purchased from a common market in the city of La Paz, Bolivia, and used in experiments where comparisons under similar environmental conditions were deemed relevant. Bantam chickens are less prone to altitude-related syndromes (pulmonary hypertension and ascites) due to their smaller size and slower growth and were preferred over meat or proper egg-laying breeds. Animals were kept in the same facilities than the Ornate Tinamous at UMSA, La Paz, Bolivia.

We also used preserved specimens from 8 tinamou species: the Great Tinamou *Tinamus major* (N = 2), the Undulated Tinamou *Crypturellus undulatus* (N = 2), the Tataupa Tinamou *Crypturellus tataupa* (N = 1), the Red-winged Tinamou *Rhynchotus rufescens* (N = 3), the Ornate Tinamou *Nothoprocta ornata* (N = 2), the Andean Tinamou *Nothoprocta pentlandii* (N = 2), the White-bellied Nothura *Nothura boraquira* (N = 2), and Darwin’s Nothura *Nothura darwinii* (N = 3). Specimens were collected for skin preservation purposes under an experimental hunting permit from the Bolivian General Direction of Biodiversity and Protected Areas (DGBAP). The bodies were preserved in ethanol 90% and deposited in the Colección Boliviana de Fauna, La Paz, Bolivia.

### Ethical considerations

Experiments in Bolivia were carried out under ethical license from the Animal Research Ethical Committee of the Bioethical National Board (CEI-CNB) issued in December 2011. Experiments in Sweden were carried out under ethical permits granted to J. Altimiras by the regional ethical committee of Linköping (Dnr.25–10, 26–10, 19–11 and 9–13). All experiments were performed in accordance with relevant guidelines and regulations.

### Heart Morphometry

Animals were killed by gunshot or euthanized by decapitation. Body mass was immediately obtained with a spring scale and the heart was dissected out, rinsed in 0.9% NaCl, immediately placed in cold cardioplegic solution (in mM: 40 NaCl, 100 KCl, 2 Ca_2_Cl, 1.8 K_2_HPO_4_, 10.1 Na_2_HPO_4_, pH = 7.4) to arrest the heart in diastole and kept in a cold environment. Within 6 h post-mortem the hearts were dissected to remove the central outflow tract, the atria and the fat deposits at the atrio-ventricular boundary and the ventricles were blotted dry before weighing on a digital scale down to 0.01 g resolution. Right ventricular mass was obtained in a subset of hearts by weighing the free right ventricular wall after dissection. The conspicuous muscular right atrioventricular valve characteristic of bird species was also included as part of the right ventricular mass.

The other subset of hearts was used to measure wall thickness by embedding the ventricles in cryo-medium (Tissue-Tek O.C.T., Sakura Finetek Europe, Leiden, the Netherlands) and freezing and cutting in a cryostat (Microm HM 550, Thermo Fisher Scientific, Walldorf, Germany) in 500 micrometer sections as previously reported^71^. Prior to freezing both ventricles were filled with a volume of cryo-medium corresponding to half of the calculated stroke volume calculated from allometric equations^72^ (SV = 0.175 HeartMass^1.05^). In preliminary tests we observed that a volume of cryo-medium equivalent to the calculated stroke volume caused the right ventricular wall to rupture at the time of freezing so the volume was halved to avoid wall rupture while keeping a clear definition of the internal chamber diameter.

All cryostat sections were photographed directly on the specimen holder of the cryostat at 20x magnification by mounting a USB Mediscope camera (Optilia Instruments AB, Sollentuna, Sweden) in the cryostat freezing chamber. Images from each heart were calibrated by placing a metallic circle of known dimensions on the specimen holder at the start and the end of each session. Particular care was placed in aligning the heart on its long axis before freezing to insure non-skewed cross sections. Right and left ventricular thickness was taken from the last two consecutive and most caudal sections in which the right atrioventricular valve (RAV) was still visible as graphically depicted in Supplementary Figure 4. Unlike in mammals, the RAV in birds is muscular^73^ and it is very apparent in cross-sections. The thickness of the right and the left free ventricular walls in each section was determined by averaging 10 single measurements that spanned the entire free wall in each section using NIS- Elements Advanced Research software (Nikon Instruments). To normalize for differences in ventricular mass all measurements of wall thickness were made relative to the diameter of the heart in the respective sections. The diameter was estimated geometrically from the total area of the section assuming a circular shape. Hearts from ethanol preserved tinamou specimens of different species were sectioned directly by hand and measurements of ventricular wall thickness were done from images in the same manner as above.

Ventricular mass data at different ages and stages of development (from embryonic age to an adult mass of 700 g) was collected from different species. Data on tinamous was collected for the purpose of this work. Data from Red Junglefowl and alligator was compiled from previous studies from our group.

### Echocardiography in conscious birds

We used portable ultrasound equipment (LogicScan 64 FLT-1T, Telemed, Vilnius, Lithuania) to image the heart in conscious birds in a right parasternal short-axis view using a 9 MHz linear probe (HL9.0/40/64D, Telemed, Vilnius, Lithuania). Wall thickness of the free left ventricular wall was measured using Echo Wave II software (Telemed, Vilnius, Lithuania) by focusing the probe on the transverse section where the right ventricular chamber was no longer apparent. We used tonic immobility as a way to avoid anesthesia and obtain data in conscious individuals. Tonic immobility was induced by restraining a bird on its back for 15 s and releasing the pressure exerted by the hand gently. Ornate Tinamous went readily into tonic immobility after one or two inductions while chickens required a maximum of five induction attempts. Although tonic immobility can be maintained for long periods and it is not harmful to the animal, it was kept only as long as needed for the procedure, typically under 5 min.

### Cardiovascular function in anesthetized animals

Anesthesia was induced in a plastic box with 4% isoflurane provided by a vaporizer (Tec 3, Ohmeda). Once the animal lost equilibrium it was placed on a heating pad with a loose plastic mask suppling the anesthetic gas mixture (1% Isoflurane:Oxygen) at a rate of 40 ml min^−1^. Ventilation rate and heart rate were continuously monitored from subcutaneous electrodes using an impedance converter (Model 2991, UFI, Morro Bay, California, USA) connected to a Powerlab amplifier (Model 4/35 ADInstruments-Europe, Oxford, UK) and the data was logged in a computer using LabChart 7Pro software (ADInstruments-Europe, Oxford, UK). Body temperature was monitored using a cloacal probe connected to the same monitoring system via a temperature pod (T-type Pod ML312, ADInstruments-Europe, Oxford, UK) and maintained at an average of 40 °C for Red Junglefowl and 39 °C for Ornate Tinamous with the use of the heating pad.

After a surgical plane of anesthesia was achieved by adjusting isoflurane concentration in the breathing mask we proceeded to catheterize the ulnar artery in the right wing with a polyethylene catheter (PE-90, 1.27 mm external diameter, 0.86 mm internal diameter, Clay-Adams Intramedic, New York, USA) pulled to a thinner tip. The tip was advanced a length of 10 mm into the artery in an upstream direction and secured in place with sutures. The catheter was then coupled to a disposable blood pressure transducer (DPT610, Peter von Berg Medizintechnik GmbH, Eglharting, Germany) connected to a bridge amplifier (FE221 ADInstruments-Europe, Oxford, UK) and to the same Powerlab amplifier and recording system. Access to the aorta to record cardiac output required the opening of the interclavicular air sac but this interfered with gas anesthesia because a stable control of inhaled isoflurane concentration could no longer be achieved. While the animal was still anesthetized with isoflurane we switched to injectable anesthesia with a mixture of ketamine (20 mg kg^−1^) and xylazine (5 mg kg^−1^) administered intraperitoneally. Once the first injection took effect we removed isoflurane from the breathing gas and we injected a second dose of anesthesia to achieve a final dose of 40:10 ketamine:xylazine. After the new anesthesia took full effect we opened the interclavicular sac, identified the aorta after the branching from the right brachiocephalic artery, freed it from connective tissue and placed a perivascular transit-time Doppler flow probe (H3MB 3 mm, Transonic Systems Inc., Ithaca, New York, USA) around it. The probe was connected to a flowmeter (T106, Transonic Systems Inc., Ithaca, New York, USA) and this to the recording system.

The whole operation up to this point would typically take 60–90 min after which the animal was monitored for stable cardiovascular parameters for at least 15 min. We later proceeded with a bolus injection of saline solution (1 ml kg^−1^) to discard volume effects from the subsequent injection of isoproterenol, a beta-adrenergic agonist, at a dose of 3 ug kg^−1^. Fifteen minutes after injection of isoproterenol the animal was euthanized by decapitation.

Blood pressure and heart rate were directly obtained from the physiological recordings. Cardiac output was estimated as 1.61 fold the measurement of aortic blood flow. This adjustment factor was obtained in a separate study in domestic chickens in which we measured blood flow in the aorta and in both brachiocephalic arteries. Brachiocephalic flow amounted to 32.6% of the total flow while coronary flow was estimated as 5.78% of the total cardiac output^74^. These validation measurements are shown in Supplementary Figure 5.

### Hematology

0.2 ml of blood were drawn from the ulnar vein in 8 males and 8 females from each of the three species while the animals were kept in resting conditions in their respective cages. Hematocrit was measured by centrifugation in standard capillary tubes, hemoglobin using a HemoCue hemoglobinometer^75^ and Red Blood Cell Count by standard counting of diluted blood in a hemocytometer. Standard equations were used to calculate mean corpuscular volume, mean corpuscular haemoglobin and mean corpuscular haemoglobin concentration.

### Metabolic and thermoregulatory response to an aerobic challenge test

For the aerobic challenge test both tinamou species and Red Junglefowl (or bantam chickens in the case of the body temperature measurements) were moved to a larger room (15–30 m^2^) where they were chased by a researcher and forced to carry out three short flapping flights and be on the run for a period of three minutes. After this time the animals were placed on their backs to trigger righting reflexes consecutively until the animals went into tonic immobility. When this occurred the challenge was concluded and we proceeded with the post-challenge measurements. The short duration of the challenge was dictated by the low stamina shown by tinamous in previous pilot runs, which correspond well to the escape behaviour described in field studies^34^. In general, tinamous prefer running to flying and only take off when pressed for it^34^. After the challenge both species of tinamous appeared unequivocally exhausted and displayed fast gular fluttering, but not Red Junglefowl or bantam chickens.

The aerobic challenge test was used in three procedures carried out separately: (1) blood lactate determination, (2) oxygen consumption measurements and (3) body temperature monitoring.

Blood lactate was measured before and immediately after the challenge test taking a small blood sample from an ulnar vein punction. The blood sample was processed for immediate determination of lactate concentration using a portable analyser (Lactate-Pro, Arkray Inc, Kyoto, Japan). The lactate analyser has been previously validated for use with bird blood^75^.

Oxygen consumption was measured by open respirometry in a push-mode configuration before and immediately after the aerobic challenge test previously described. The animal was placed in a 6 liter air-tight chamber (18 cm diameter × 25 cm height) equipped with two sets of tubing (Tygon R3603 3.2 × 4.8 mm) leading air in and out with a controlled flow of 1200 ml min^−1^ (FOX II Analyzer, Sable Systems International, Las Vegas, USA). To avoid dilution effects by the presence of water vapor, the air sample was dried through a desiccator column (30 ml of indicating drierite, anhydrous calcium sulfate mixed with cobalt chloride, W. A. Hammond Drierite company Ltd, Xenia, USA). The FOX II Analyzer was connected to a laptop computer (Dell Latitude D600, Dell Inc., Round Rock, Texas, USA) via a serial connection and that data was stored using a custom made data acquisition program (Lab View 8.6, National Instruments, Austin, Texas, USA). Chamber flow (1200 ml min^−1^) was set according to chamber volume and predicted VO_2_ using published recommendations. Oxygen consumption was calculated using standard equations for the case when water vapor but not CO_2_ is stripped from the gas sample as follows: V_O2_ = Flow x ([O_2_]in-[O_2_]out) / (1-0.2 x [O_2_]out) where [O_2_]in and [O_2_]out are the concentrations of oxygen entering and exiting the chamber respectively.

Body temperature was measured using a T-type thermocouple (RET-2, MLT1403, ADInstruments-Europe, Oxford, UK) inserted in the cloaca an average length of 55 mm and the leading wire was fixed with tape to the root of a tail feather. The thermocouple was connected to a temperature pod (T-type Pod ML312, ADInstruments-Europe, Oxford, UK), a Powerlab amplifier (Model 4/35 ADInstruments-Europe, Oxford, UK) and finally the data was logged in a computer using LabChart 7Pro software (ADInstruments-Europe, Oxford, UK). To account for the effect of circadian rhythms all measurements were carried out between 10.00 and 15.00. A bird was taken from its cage without struggle in resting conditions and placed in 6-L plastic containers (18 cm diameter × 25 cm height) for a period of 3 h (pre-challenge baseline). After the aerobic challenge the animal was re-instrumented with the cloacal probe and immediately returned to the holding container for subsequent measurements (post-challenge, 2 h). Measurements were carried out at two different ambient temperatures, at 25 °C (range 23.2–27 °C) in an incubator (Yonar Incubadoras, Buenos Aires, Argentina) or 5 °C (range 3.8–5.6 °C) in a refrigerator (FR093R, Daewoo Electronics Corp., Seoul, South Korea). Ambient temperatures in the enclosures at the time the measurements were made varied between 10–15 °C.

### Gene expression

Myocardial tissue from the three species was obtained post-mortem and preserved in RNA*later*® Tissue Collection: RNA stabilization solution (Applied Biosystems, Thermo Fisher Scientific, Walldorf, Germany) for 48 h at 4 °C and later at −80 °C prior to tissue processing. Total RNA was isolated using TRIzol reagent (Thermo Fisher Scientific, Walldorf, Germany) and reverse transcribed into cDNA using Revert Aid H Minus First strand cDNA synthesis kit with Oligo(dT)_18_ primers (Fermentas, Burlington, ON, Canada). Quantitative real-time PCR was carried out using the Roche Light-cycler 480 (Roche Applied Science, Roche Diagnostics, Basel, Switzerland) and Maxima SYBR Green qPCR master mix (Fermentas, Burlington, ON, Canada). Levels of *ERK2*, *p38*, *JNK1* and *PIK3CA* transcripts were normalized to the expression of *TBP*, *ACTB* and *GAPDH* using the ΔCt-method. Gene nomenclature and specific primers are provided in Supplementary Table 2.

### Statistical Analysis

All results are presented as average with standard deviations (s.d.). Statistical analysis was carried out using general linear models followed by posthoc Tukey tests (Minitab v.17, MiniTab Inc, State College, PA, USA) or permutation tests (StatBoss permutation tester, M.J.Lew, Department of Pharmacology, The University of Melbourne,^76^). Permutation tests are adequate and more robust than parametric tests to compare differences between groups^76^.

Power regression analysis on cardiac growth for the different species was carried out after double logarithm transformation and Model II regression analysis (orthogonal regression) in Minitab (v.17, MiniTab Inc, State College, PA, USA). Model II regression was preferred over Model I regression because both variables (body mass and ventricular mass) are obtained experimentally and include random measurement error^77^.

Specific details on statistical procedures are detailed in each figure legend.

## Electronic supplementary material

Supplementary Material
