# Supplementary material for: Aerobic performance in tinamous is limited by their small heart. A novel hypothesis in the evolution of avian flight
Source: Sci Rep. 2017 Nov 21;7:15964. doi: 10.1038/s41598-017-16297-2 (PMC5698454; doi:10.1038/s41598-017-16297-2)
Supplement: Supplementary file 1 — Supplementary Material [file 41598_2017_16297_MOESM1_ESM.pdf]

SUPPLEMENTARY INFORMATION to:

"Aerobic performance in tinamous is limited by their small heart. A novel hypothesis in the evolution of avian flight" by Jordi Altimiras, Isa Lindgren, Lina María Giraldo-Deck, Alberto Matthei and Álvaro Garitano-Zavala

Supplementary Material and Methods

The data for Suppl.Figure 1 is from a compilation of heart and body mass data for adult birds through an extensive literature search in the Zoological Record (Thomson Reuters). Details on the compilation of the data can be obtained from our companion study (*1*). The full dataset of average heart mass and body mass per species, sex from the different scientific studies is available online (http://www.ifm.liu.se/biology/zoology/avian/staff/altimiras/birdheartdatabase/index.xml).

We obtained a pondered average heart mass per species by pooling data from males and females if available and pooling data from different studies. The pondered average was applied to give more weight to measurements obtained from multiple individuals than studies in which only one specimen was measured. We used the Jetz phylogeny to group all species in families (*2*). A family average was obtained for the bird families represented by nine or more species. Because of its relevance to the discussion we also grouped together three species in the superorder Ratites, the Ostrich, the Greater Rhea and the Emu.

References

1. R. F. Nespolo, C. González-Lagos, J. J. Solano-Iguaran, M. Elfwing, A. Garitano-Zavala, S. Mañosa, J. C. Alonso, J. Altimiras, The adaptive evolution of flight mode and aerobic power in birds: a phylogenetic test of the heart-size hypothesis. *J.exp.Biol.* **resubmitted after review** (2017).
2. W. Jetz, G. H. Thomas, J. B. Joy, K. Hartmann, A. O. Mooers, The global diversity of birds in space and time. *Nature* **491**, 444-448 (2012).

Supplementary Table 1

Hematological variables measured in males (N=8) and females (N=8) from the Red Junglefowl and the two tinamou species. Data shown as Mean (standard deviation). Dissimilar letters indicate significant differences assessed by Tukey posthoc tests following one way ANOVA.

**Species**	**Sex**	**[Hb]** **($g\ L^{-1}$)**	**Ht** **(%)**	**RBC** **($x10^6$ cells μl^{-1})**	**MCV** **(fl)**	**MCH** **(pg)**	**MCHC** **($g\ dL^{-1}$)**
Gallus gallus	Female	107.5 (8.0) ^{D}	35.8 (2.7) ^{B}	2.53 (0.23) ^{B}	141.6 (8.1) ^{B}	42.6 (3.1) ^{C}	30.1 (1.0) ^{B}
	Male	151.9 (9.6) ^{A}	50.2 (3.8) ^{A}	3.19 (0.27) ^{A}	157.6 (8.0) ^{B}	47.7 (2.1) ^{C}	30.3 (0.6) ^{B}
Nothoprocta perdicaria	Female	123.0 (2.1) ^{BC}	39.2 (1.0) ^{B}	2.13 (0.16) ^{BC}	184.7 (15.9) ^{A}	57.9 (3.7) ^{AB}	31.4 (1.0) ^{B}
	Male	119.9 (5.3) ^{CD}	37.9 (1.9) ^{B}	2.11 (0.16) ^{C}	180.4 (9.6) ^{A}	57.1 (3.1) ^{B}	31.6 (1.0) ^{B}
Nothoprocta ornata	Female	128.4 (6.6) ^{BC}	37.1 (2.2) ^{B}	2.36 (0.25) ^{BC}	158.8 (17.6) ^{B}	55.0 (6.0) ^{B}	34.7 (1.4) ^{A}
	Male	135.4 (11.7) ^{B}	38.2 (4.1) ^{B}	2.13 (0.32) ^{C}	180.8 (12.8) ^{A}	64.4 (7.3) ^{A}	35.6 (2.5) ^{A}

Supplementary Table 2

Gene identities, sequence IDs and primer sequences used in the analysis of gene expression displayed in Figure 7

**Gene Name**	**Description**	**Sequence ID**	**Forward Primer**	**Reverse Primer**
ERK2	*Gallus gallus* mitogen-activated protein kinase 1 (MAPK1), mRNA	NM_204150.1	CCAATGTGCTTCAT CGCGACCT	CTGCAACACGAGC CAGTCCG
JNK1	*Gallus gallus* mitogen-activated protein kinase 8 (MAPK8), predicted mRNA	Transcripts X1-X6 XM_015288441.1 XM_004942133.2 XM_015288440.1 XM_015288439.1 XM_001233168.4 XM_004942132.2	GGCTGGGAACAGA ATTTGGATG	ATTGTTGTCACGCT TGCTTCT
P38	*Gallus gallus* mitogen-activated protein kinase 14 (MAPK14), predicted mRNA	Transcripts X1-X2 XM_419263.4 XM_001232615.2	AGTGGGATGCATTA TGGCTGA	GGGGTTCCAACGA GTCTCAA
PIK3CA	*Gallus gallus* phosphoinositide 3-kinase catalytic subunit, mRNA	NM_001004410.1	CTGCGGGGAAAGC GAGATGGA	CCATCCACCACAA CAGAGCAGGC
TBP	*Gallus gallus* TATA-box binding protein , mRNA	NM_205103.1	GAACCACGTACTAC TGCGCT	GCCAGTCTGGACT GTTCCTC
ACTB	*Gallus gallus* Actin, beta, mRNA	NM_205518.1	CACAGATCATGTTT GAGACCTT	CATCACAATACCA GTGGTACG
GAPDH	*Gallus gallus* Glyceraldehyde-3-phosphate dehydrogenase, mRNA	NM_204305.1	GTCAAGGCTGAGA ACGGGAA	GCCCATTTGATGTT GCTGGG

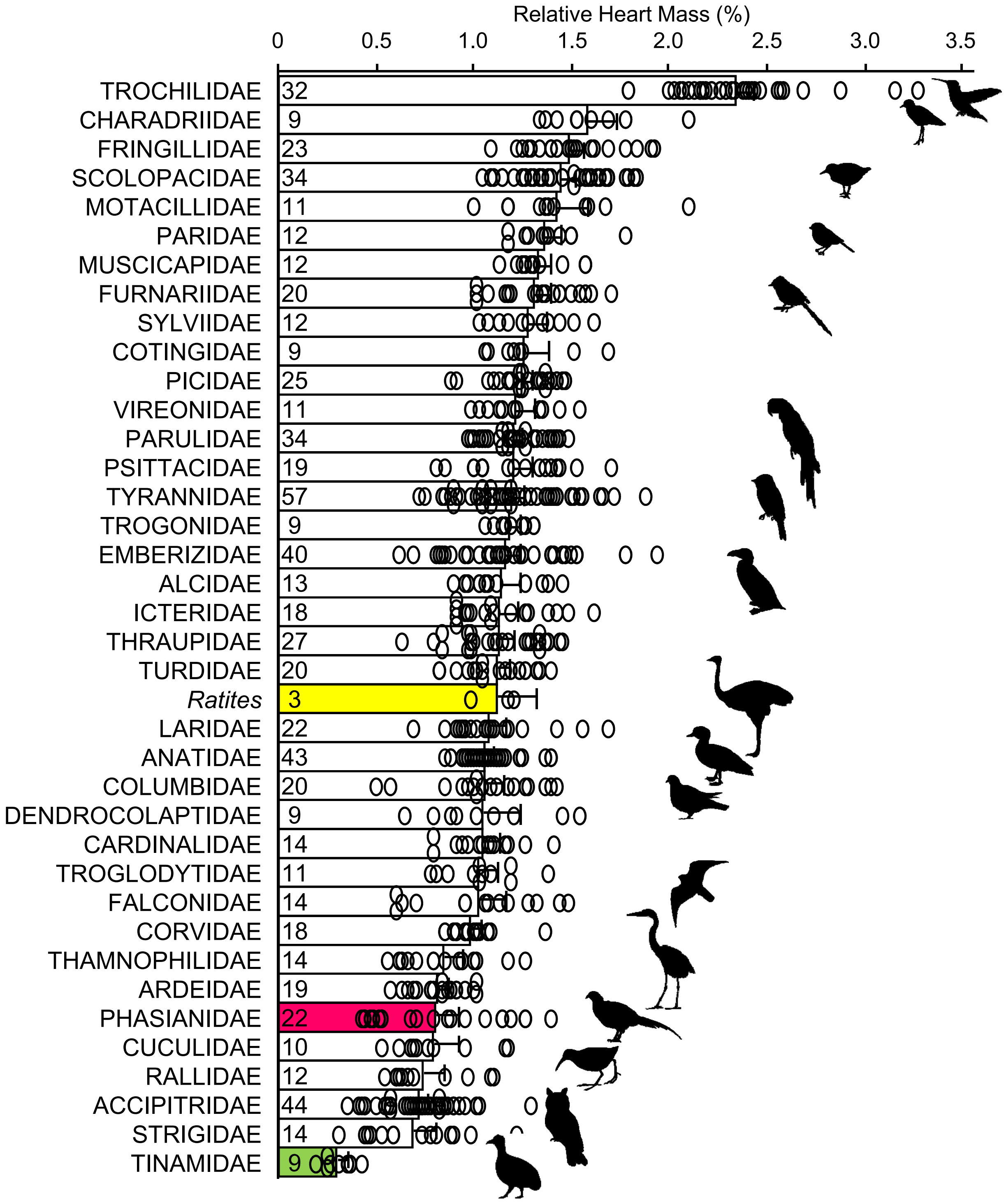

Supplementary Figure 1

Average relative heart mass in different bird families. Families shown are those represented by at least nine different species. Highlighted in color are the families Tinamidae (in green) and Phasianidae (in red, which includes the Red Junglefowl) and the superorder Ratites (in yellow), which is shown for its relevance to the discussion The number of species included in each family is shown at the base of the bar. Data shown as average and 95% confidence intervals, with the individual data points per species shown. Further details provided in Supplementary Material and Methods.

Complete Legend to Supplementary Figure 1

Average relative heart mass in different bird families. Families shown are those represented by at least nine different species. Highlighted in color are the families Tinamidae (in green) and Phasianidae (in red, which includes the Red Junglefowl) and the superorder Ratites (in yellow), which is shown for its relevance to the discussion The number of species included in each family is shown at the base of the bar. Data shown as average and 95% confidence intervals, with the individual data points per species shown. Further details provided in Supplementary Material and Methods. Some bird silhouettes were obtained from Phylopic.org and among them some require special credit. From top to bottom when due: Charadriidae and Anatidae - images provided by Rebecca Groom under the Creative Commons Attribution 3.0 Unported license (https://creativecommons.org/licenses/by/3.0/); Scolopacidae - image provided by Elaine R.Wilson (modified) under the Creative Commons Attribution-ShareAlike 3.0 Unported license (https://creativecommons.org/licenses/by-sa/3.0/); Tyrannidae - image provided by Emily Willoughby under the Creative Commons Attribution-ShareAlike 3.0 Unported license (https://creativecommons.org/licenses/by-sa/3.0/); Ratites - image provided by Matt Martyniuk (vectorized by T.Michael Keesey under the Creative Commons Attribution-ShareAlike 3.0 Unported license (https://creativecommons.org/licenses/by-sa/3.0/); Columbidae - Image provided by Dori dori@merr.info (source photo) and Nevit Dilmen under the Creative Commons Attribution 3.0 Unported license (https://creativecommons.org/licenses/by/3.0/) and Falconidae - Image provided by Liftarn under the Creative Commons Attribution-ShareAlike 3.0 Unported license (https://creativecommons.org/licenses/by-sa/3.0/).

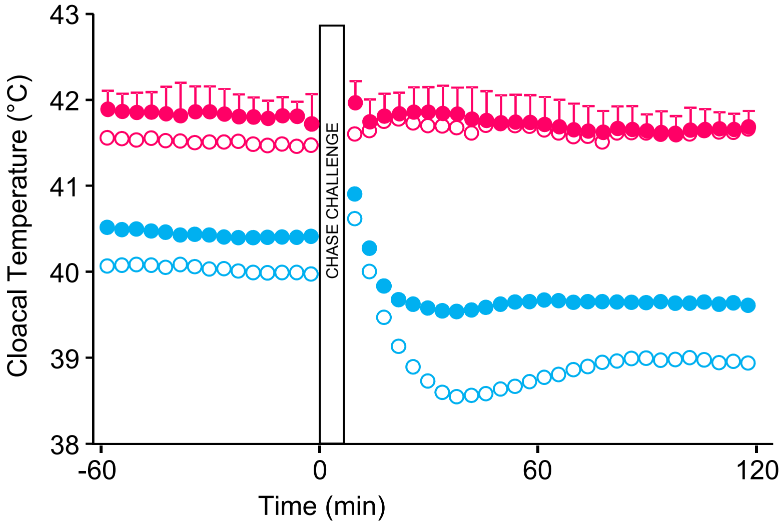

Supplementary Figure 2 (completes Figure 6 from the article)

Cloacal temperature before and after a 3 min chase-and-exhaust protocol in Ornate Tinamou (blue symbols) and domestic chickens (red symbols) kept at an ambient temperature of 4°C (open symbols) and 25°C (closed symbols). The chase-and-exhaust protocol was carried out after a baseline measurement lasting 1 h. Data from chickens at 25°C is presented fully and the data already shown in paper Figure 6 is displayed for comparison without standard deviations. No significant changes in cloacal temperature were observed in chickens at 25°C.

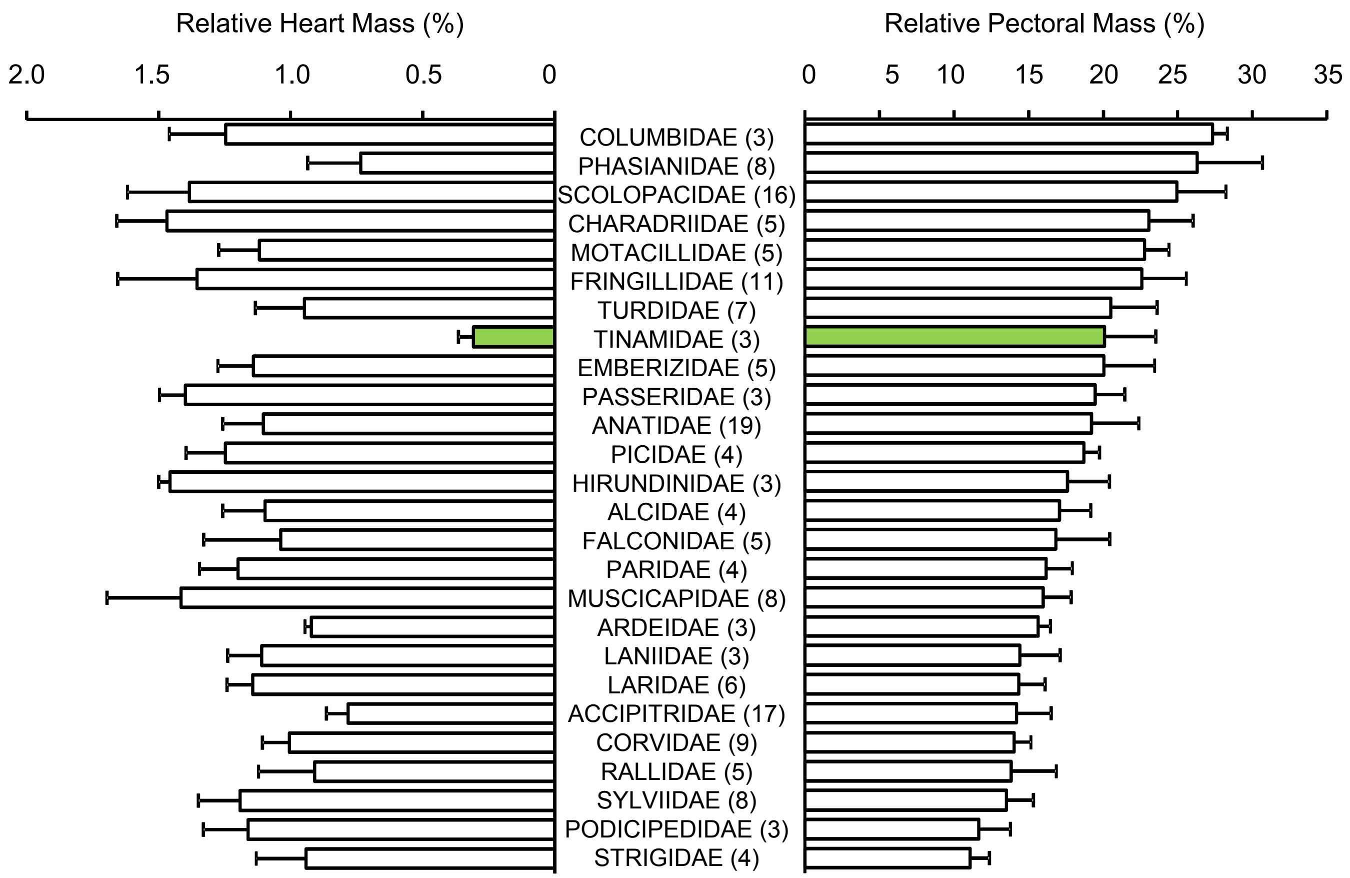

Supplementary Figure 3

Average relative heart mass (bars on the left) and relative pectoral mass (bars on the right) in different bird families represented by three or more species. Data obtained Magnan 1922 except data from Chilean and Ornate Tinamous, which are from our own measurements. Data shown as average and standard deviations. The number of species included in each family is shown in parenthesis.

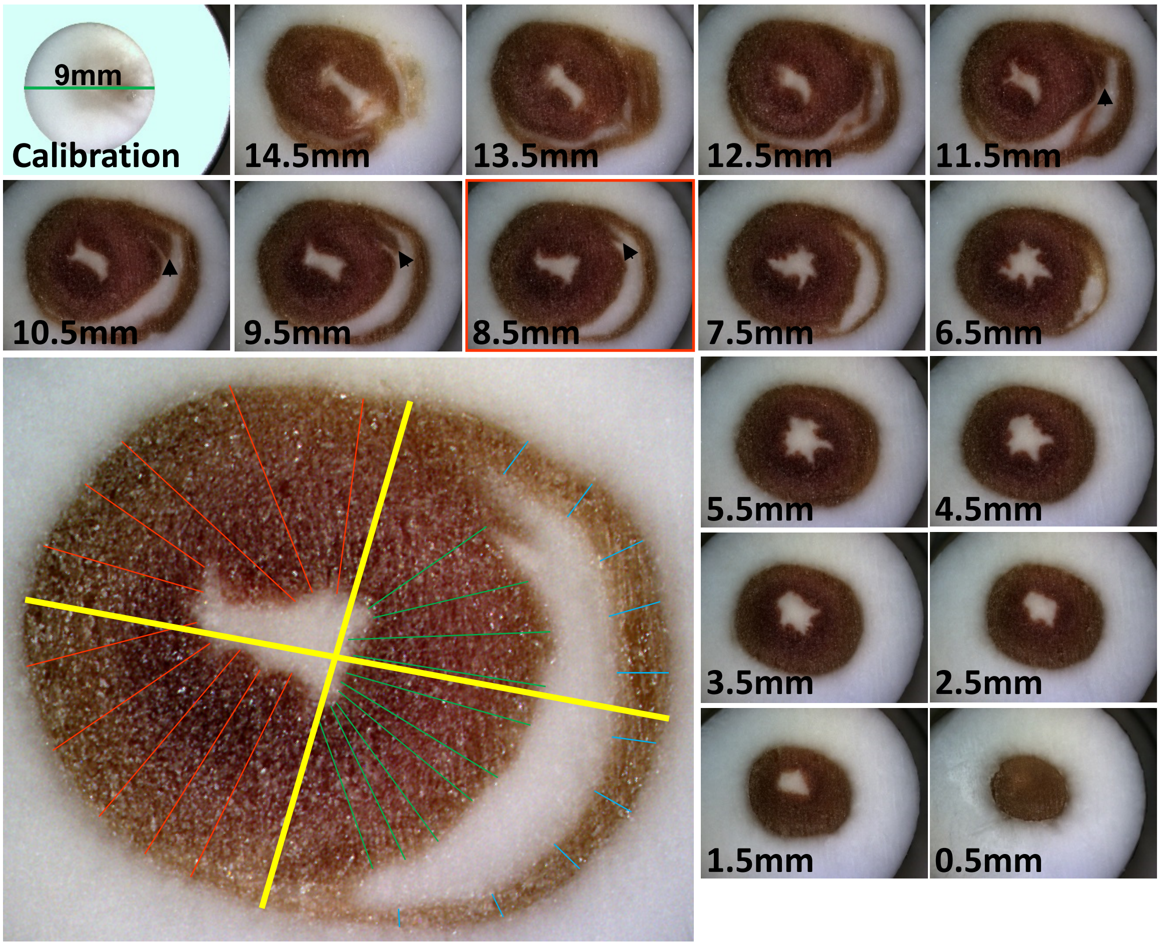

Supplementary Figure 4

Pictorial description of the method for measuring ventricular wall thickness in the heart of a Chilean Tinamou *Nothoprocta perdicaria*. Sectioning was done in 500 um sections but only every other section is presented to show the entire heart together with one calibration picture. The sections used for analysis were two consecutive sections (only one shown, 8.5 mm from the apex of the heart) in which the right atrioventricular valve (RAVV) was still visible. Tip of the RAVV is shown by arrowheads. Notice that the RAVV becomes a muscular band with attachments on the ventral and dorsal side of the heart when closer to the base (seen in the sections 11.5 and 12.5 mm from the apex). The following measurements were taken: long and short axis (yellow lines) and 10 equidistributed measurement of the ventricular walls: free left ventricular wall (red lines), septal wall (green lines) and free right ventricular wall (blue lines). Free left ventricular wall and septal wall were averaged to represent the thickness of the left ventricle.

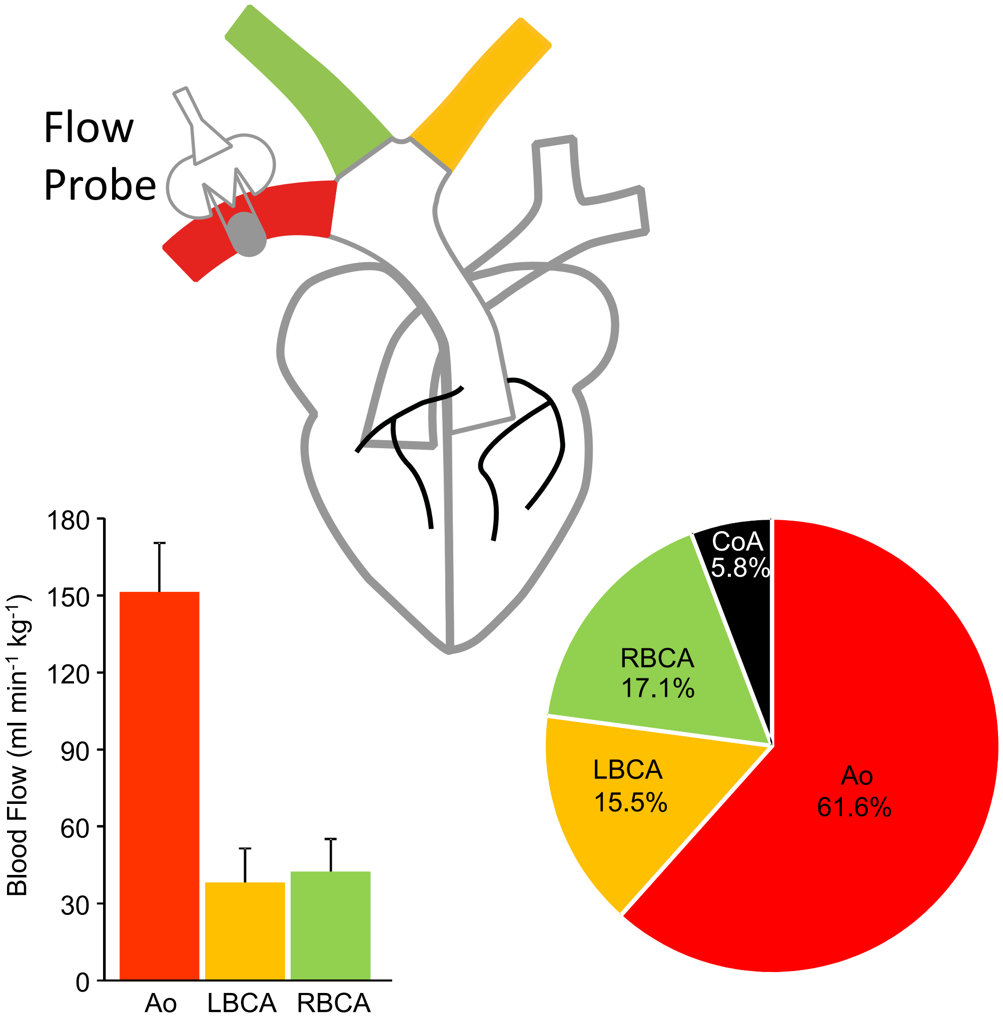

Supplementary Figure 5

Blood flow measurements in domestic chickens (N=4) carried out to estimate the contribution of brachiocephalic flow to total cardiac output. Flow in the left and right brachiocephalic arteries (LBCA and RBCA respectively) were measured in the same individuals after measuring flow in the aorta (Ao). Values shown as means and standard deviations. Total flows shown on the left graph. Relative flows (% of cardiac output) shown on the chart on the right. Flow in the coronary artery (CoA) was estimated as 5.8% of the total flow based on literature values in chickens. This value is likely to overestimate coronary flows in tinamous with smaller hearts, which will make the cardiac output measurements more conservative. Based on these measurements, the aortic flow values from the main study were corrected to account for brachiocephalic and coronary flows.
